# The effect of exercise training on clinical outcomes in patients with the metabolic syndrome: a systematic review and meta-analysis

**DOI:** 10.1186/s12933-017-0590-y

**Published:** 2017-08-30

**Authors:** C. Ostman, N. A. Smart, D. Morcos, A. Duller, W. Ridley, D. Jewiss

**Affiliations:** 1 School of Rural Medicine, University of New England, Armidale, NSW 2351 Australia; 2 School of Science and Technology, University of New England, Armidale, NSW 2351 Australia

**Keywords:** Metabolic syndrome, Exercise training, Meta-analysis

## Abstract

**Background:**

*Purpose*: to establish if exercise training improves clinical outcomes in people with metabolic syndrome (MetS). Registered with PROSPERO international prospective register of systematic reviews (https://www.crd.york.ac.uk/PROSPERO/Identifier:CRD42017055491). *Data sources*: studies were identified through a MEDLINE search strategy (1985 to Jan 12, 2017), Cochrane controlled trials registry, CINAHL and SPORTDiscus. *Study selection*: prospective randomized or controlled trials of exercise training in humans with metabolic syndrome, lasting 12 weeks or more.

**Results:**

We included 16 studies with 23 intervention groups; 77,000 patient-hours of exercise training. In analyses of aerobic exercise studies versus control: body mass index was significantly reduced, mean difference (MD) −0.29 (kg m^−2^) (95% CI −0.44, −0.15, p < 0.0001); body mass was significantly reduced, MD −1.16 kg (95% CI −1.83, −0.48, p = 0.0008); waist circumference was significantly reduced MD −1.37 cm (95% CI −2.02, −0.71, p < 0.0001), peak VO_2_ was significantly improved MD 3.00 mL kg^−1^ min^−1^ (95% CI 1.92, 4.08, p < 0.000001); systolic blood pressure and diastolic blood pressure were significantly reduced, MD −2.54 mmHg (95% CI −4.34, −0.75, p = 0.006), and, MD −2.27 mmHg (95% CI −3.47, −1.06, p = 0.0002) respectively; fasting blood glucose was significantly reduced MD −0.16 mmol L^−1^ (95% CI −0.32, −0.01, p = 0.04); triglycerides were significantly reduced MD −0.21 mmol L^−1^ (95% CI −0.29, −0.13, p < 0.00001); and low density lipoprotein was significantly reduced MD −0.03 mmol L^−1^ (95% CI −0.05, −0.00, p = 0.02). In analyses of combined exercise versus control: waist circumference, MD −3.80 cm (95% CI −5.65, −1.95, p < 0.0001); peak VO_2_ MD 4.64 mL kg^−1^ min^−1^ (95% CI 2.42, 6.87, p < 0.0001); systolic blood pressure MD −3.79 mmHg (95% CI −6.18, −1.40, p = 0.002); and high density lipoprotein (HDL) MD 0.14 (95% CI 0.04, 0.25, p = 0.009) were all significantly improved. We found no significant differences between outcome measures between the two exercise interventions.

**Conclusions:**

Exercise training improves body composition, cardiovascular, and, metabolic outcomes in people with metabolic syndrome. For some outcome measures, isolated aerobic exercise appears optimal.

**Electronic supplementary material:**

The online version of this article (doi:10.1186/s12933-017-0590-y) contains supplementary material, which is available to authorized users.

## Introduction

According to the international diabetes federation, the metabolic syndrome (MetS) is characterised by at least three of five clinical findings: central obesity, elevated blood pressure (systolic blood pressure ≥130 mmHg or diastolic blood pressure ≥85 mmHg), elevated serum triglycerides (≥150 mg dL^−1^), low serum high-density lipoprotein (HDL) (<40 mg dL^−1^in males and <50 mg dL^−1^ in females), and insulin resistance (fasting plasma glucose ≥100 mg dL^−1^) [1]. More than 20% of the world’s population is estimated to meet the diagnostic criteria for MetS, and are thus three times more likely to develop cardiovascular disease and five times more likely to develop type 2 diabetes, presenting an enormous public health issue [2].

Previous data pooling analyses are yet to show a wide scope of benefit from exercise training in people with metabolic syndrome. Analyses of lifestyle interventions are yet to show a reduction in the proportion of participants who meet the diagnostic criteria for MetS [3], although summary data are available outlining significant improvements in both body composition and metabolic profile measures with lifestyle intervention in women with metabolic derangement due to polycystic ovarian syndrome [4, 5]. Lifestyle (diet and exercise) has been shown to be effective in resolving MetS and reducing the severity of related abnormalities (fasting blood glucose, waist circumference, systolic blood pressure and diastolic blood pressure, and triglycerides) [3]. While the beneficial clinical effects of lifestyle interventions have been shown in meta-analyses of participants with type II diabetes [6], the relative contribution of exercise, in deriving these benefits, is unknown. Resistance training interventions of patients with MetS has been found to reduce systolic blood pressure, but not fasting plasma glucose, HDL cholesterol, triglycerides, diastolic blood pressure, or waist circumference [7]. It is however possible that the small number of included studies precluded significant improvements in other outcome relevant measures. The meta-analysis by Aguilera et al. [8] was unable to uncover enough evidence (only one study yielded eligible data) to draw meaningful conclusions around the optimal exercise intensity to treat metabolic syndrome. The work of Lin et al. [9] reported beneficial effects of exercise training on cardiac risk factors, but this work included studies of both healthy people and those with metabolic disease. It remains unclear which type of exercise, and at what intensity, is optimal for patients.

We therefore conducted a systematic analysis of all clinical randomized, controlled, exercise training trials in patients with MetS and stratified the trials by exercise intensity according to recognized guidelines. We aimed to determine whether high-intensity exercise produced different effect sizes for change in clinical outcomes in MetS compared to vigorous-, moderate- and low-intensity training and sedentary lifestyle. We also wished to establish whether the effect on clinical outcomes in MetS varied according to the type of intervention (aerobic versus combined aerobic and resistance training).

## Methods

The review protocol was registered in PROSPERO international prospective register of systematic reviews (https://www.crd.york.ac.uk/PROSPERO/Identifier:CRD42017055491).

### Data sources and search strategy

Studies were identified through a MEDLINE search strategy (1985 to Jan 12, 2017), Cochrane controlled trials registry (1966 to Jan 12, 2017), CINAHL, SPORTDiscus and science citation index. The search strategy included a mix of MeSH and free text terms for key concepts related to exercise training and the metabolic syndrome (see PubMed search strategy in Additional file 1). Studies were included if patients in the intervention group met the diagnostic criteria for metabolic syndrome (according to IDF, WHO, or NCEP-ATP III) [1]. Searches were limited to prospective randomized or controlled trials of exercise training in humans. Only English language studies were included. No restrictions were placed on the year of publication. Reference lists of papers were scrutinised for new articles. Full articles were read and assessed by two reviewers (CO and DJ) for relevance and study eligibility. Disagreements on methodology were resolved by discussion, and a third reviewer (NS) adjudicated over any disputes. Study authors were contacted and requested to provide further data if required.

### Study selection

Included studies were randomized controlled trials of exercise training in people with the metabolic syndrome diagnosed according to recognized diagnostic criteria (IFD, WHO, or NCEP-ATP III) [1]. All published studies included in this systematic review were comparisons between intervention groups and a sedentary control. After initial screening, we removed over-lapping and duplicate articles, as well as articles that did not meet inclusion criteria. We excluded studies whereby not all participants in the intervention group met the diagnostic criteria for metabolic syndrome at the start of the intervention, studies that did not have a sedentary control group, and those reporting only acute exercise testing responses. Studies with diet or medical interventions were included only if the intervention was constant across the exercise and control groups (Table 1). Only the principal study with the greatest number of subjects was included where multiple publications existed from the same dataset. We excluded data from specific analysis if incomplete data was reported and the authors did not respond to our requests to provide missing data.

**Table 1:** Table of included studies

Article	Country	Category	Aerobic exercise intensity	Participants	Intervention
Balducci 2010 small	Italy	Aerobic and combined	Moderate and vigorous	82 subjects with T2DM and MetS according to IDF were enrolled. They were randomised into control (n = 20), moderate intensity aerobic (n = 20), vigorous aerobic (n = 20) and combined aerobic and resistance (n = 22)	Sessions consisted of the allocated exercise for 60 min, 3 sessions per week for 52 weeks
Balducci 2010 large	Italy	Combined	Moderate	606 subjects with MetS were included. They were randomised into control (n = 303) and combined aerobic and resistance exercise (n = 303)	Sessions consisted of 75 min of combined aerobic and resistance exercise, 2 sessions per week for 52 weeks
Donley 2014	USA	Aerobic	Vigorous to high	21 healthy and 22 MetS were included. MetS participants were allocated to aerobic exercise	Sessions consisted of 60 min of aerobic exercise, 3 times per week for 8 weeks
Irving 2008	USA	Aerobic	Moderate and vigorous	37 women with MetS were included. They were randomised into control (n = 9), moderate intensity exercise (n = 15) or high intensity exercise (n = 12)	Sessions consisted of 50 min of aerobic exercise, 5 sessions per week for 16 weeks
Irving 2009	USA	Aerobic	Moderate and vigorous	34 adults with MetS were included. They were randomised into control (n = 10), moderate intensity exercise (n = 13) or high intensity exercise (n = 11)	Sessions consisted of 60 min of aerobic exercise, 5 sessions per week for 16 weeks
Kim 2011	Korea	Aerobic	Moderate	43 patients with T2DM and MeTS were randomised into control (n = 22) and aerobic exercise (n = 21)	The intervention consisted of 150 min of aerobic exercise per week for 16 weeks
Mager 2008	Finland	Aerobic	Moderate	75 patients with MetS were included. They were randomised into diet alone (n = 28), resistance training (n = 14), aerobic training (n = 15) and control (n = 18)	Sessions consisted 30 min of the allocated exercise, 2–3 times per week for 33 weeks
Maresca 2013	Italy	Aerobic	Vigorous	20 male patients with MetS were included. They were randomised into Aerobic exercise + tadalafil (n = 10) or tadalafil alone (n = 10)	Sessions consisted of 40 min of aerobic exercise, 3 times per week for 2 months
Oh 2010	Korea	Combined	Light	52 women with MetS were included. They were randomised into a combined aerobic and resistance exercise group (n = 31) or the control group (n = 21)	Sessions consisted of 40 min of combined aerobic and resistance exercise, 2–3 times per week for 6 months
Okura 2007	Japan	Aerobic	Vigorous	67 patients with MetS were included. There were allocated into low calorie diet (n = 24) or diet + aerobic exercise (n = 43)	Sessions consisted of 45 min of aerobic exercise, 3 times per week for 14 weeks
Reseland 2001	Norway	Aerobic	Moderate	186 men with MetS were included. They were allocated into diet alone (n = 44), diet + aerobic exercise (n = 57), exercise alone (n = 48) or control (n = 37)	Sessions consisted of 60 min of aerobic exercise, 3 times per week for 1 year
Soares 2014	Brazil	Aerobic	Moderate to Vigorous	87 participants with Mets were included. They were randomised into dietary intervention (n = 24), dietary intervention + exercise (n = 17), dietary intervention + omega 3 supplementation (n = 23) or dietary intervention + omega 3 supplementation + exercise (n = 23)	Sessions consisted of 30 min of aerobic exercise, 3 times per week for 12 weeks
Sonnenschein 2011	Germany	Aerobic	Moderate to Vigorous	24 subjects with MetS were included. They were randomised into aerobic exercise (n = 12) or control (n = 12)	Sessions consisted of 30 min of aerobic exercise, 5 times per week for 8 weeks
Straznicky 2010	Australia	Aerobic	Moderate	58 MetS subjects were included. The were randomised into low calorie diet (n = 20), diet + exercise (n = 20) or control (n = 19)	Sessions consisted of 40 min of aerobic exercise, 3–4 times per week for 12 weeks
Stensvold 2010	Norway	Aerobic and Combined	High	43 patients with MetS were included. They were randomised into aerobic interval training (n = 11), resistance training (n = 11), combination aerobic and resistance (n = 10) and control (n = 11)	Sessions consisted of 40 to 50 min of the allocated exercise, 3 times per week for 12 weeks
Tjonna 2008	Norway	Aerobic	Moderate	32 participants with the MetS were included. They were randomised into aerobic interval training (n = 12), continuous moderate exercise (n = 10) or control (n = 10)	Sessions consisted of 40 min of the allocated exercise, 3 times per week for 16 weeks

### Outcome measures

We recorded the following data: incident mortality and hospitalisation. We also recorded change in (baseline versus post intervention): peak VO_2_, BMI, body weight, waist circumference, systolic blood pressure, diastolic blood pressure, fasting blood glucose, fasting insulin, HOMA-IR, HbA1c%, HDL, LDL, TG, and, total cholesterol. We also recorded exercise training frequency, intensity, duration per-session, length of exercise programs and concurrent interventions e.g. diet.

### Data synthesis

From extracted data, we calculated patient-hours of exercise training, mean difference change in outcome measures, drop out and attendance rates, and medical events.

### Assessment of study quality

We assessed study quality with regards to: eligibility criteria specified, random allocation of participants, allocation concealed, similarity of groups at baseline, assessors blinded, outcome measures assessed in 85% of participants and intention to treat analysis. The study quality was assessed according to the validated TESTex scale which has a maximum score of 15 [10].

### Data analysis

Revman 5.3 (Nordic Cochrane Centre, Denmark) was used to complete the meta-analysis and generate forest plots. Pooled data are presented as mean differences. We chose a random effects model as an element of randomness is inevitable when pooling data from individual studies. A minimum of two studies was required for forest plots. Some studies used more than one intervention group, but the same people were only represented once in our forest plots.

Meta-analyses were completed for continuous data by using the change in the mean and standard deviation of outcome measures. It is an accepted practice to only use post-intervention data for meta-analysis but this method assumes that random allocation of participants always creates intervention groups matched at baseline for age, disease severity, etc. Change in post intervention mean was calculated by subtracting baseline from post-intervention values [11]. Data required was either: (i) 95% confidence interval data for pre- and post-intervention change for each group, or when this was unavailable; (ii) actual p values for pre- and post-intervention change for each group, or if only the level of statistical significance was available; (iii) we used default p values, e.g. p < 0.05 becomes p = 0.049, p < 0.01 becomes p = 0.0099 and p = not significant becomes p = 0.05. If 95% confidence intervals overlapped between two or more sub-analyses of the same outcome measure, where data was presented as ‘MD (95% CI)’, we assumed that there was no statistically significant difference between the groups.

### Heterogeneity

Heterogeneity was quantified using the I^2^ test, as it does not inherently depend on the number of studies considered [12]. I^2^ values range from 0% (homogeneous) to 100% (greater heterogeneity); a CI that does not include 0% indicates that the hypothesis of homogeneity is rejected, and an inference of heterogeneity is merited. We used a random effects model for all analyses.

### Publication bias

Funnel plots were examined for evidence of publication bias [13].

## Results

**Fig. 1:**
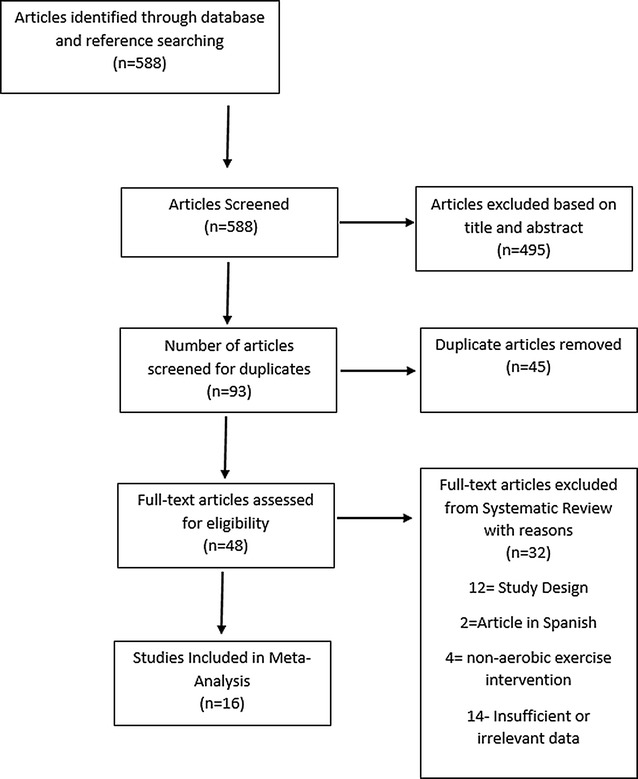
Consort statement

Records were initially identified through database searching and additional records from the reference list were added. Only the principal study with the greatest subjects was included where multiple publications existed from the same dataset. After initial screening of titles, irrelevant studies were removed, which include over-lapping studies, abstracts, and irrelevant articles, such as editorials and discussion papers that did not match the inclusion criteria. Forty-five duplicate papers were identified and removed. We excluded a further 32 studies with reasons: 12 studies due to study design, two due to the article being in Spanish, four did not have an aerobic intervention and 14 had insufficient or irrelevant data for inclusion in the meta-analysis (see consort statement, Fig. 1).

The 16 included studies [14–26] produced 23 intervention groups (See Table 1). There were 14 studies that had intervention groups that compared aerobic exercise versus sedentary control and four studies that had intervention groups that compared a combined aerobic and resistance exercise versus sedentary control. Overall, the 16 studies provided more than 800 exercising participants which resulted in more than 77,000 patient-hours of exercise training.

### Comparison of all aerobic exercise studies versus control

#### Body composition outcome measures

BMI was significantly reduced by a mean difference (MD) of −0.29 (kg m^−2^) (95% CI −0.44, −0.15, p < 0.0001) in exercise versus control groups. Body mass was significantly reduced by a mean difference of −1.16 kg (95% CI −1.83, −0.48, p = 0.0008) in exercise versus control groups. Waist circumference was significantly reduced by −1.37 cm (95% CI −2.02, −0.71, p < 0.0001) in exercise versus control groups (Table 2). Total body fat mass was significantly reduced MD −1.19 kg (95% CI −1.79, −0.59, p = 0.0001) in exercise versus control.

**Table 2:** Effect of different exercise training programs on MetS

Outcome	Aerobic exercise versus control				Combined aerobic and resistance exercise versus control			
	No. studies	N	MD (95% CI), p value	I^2^ (%)	No. studies	N	MD (95% CI), p value	I^2^
BMI (kg m^−2^)	14	385	−0.29 (−0.44, −0.15) p < 0.0001	79	3	652	−0.40 (−0.88, 0.07) p = 0.10	100%
Body weight (kg)	11	217	−1.16 (−1.83, −0.48) p = 0.0008	43	2	46	−0.03 (−0.51, 0.46) p = 0.92	24%
Waist (cm)	13	261	−1.37 (−2.02, −0.71) p < 0.0001	61	3	652	−3.80 (−5.65, −1.95) p < 0.0001	59%
Total fat mass (kg)	5	176	−1.19 kg (−1.79,−0.59) = 0.0001	0	0	0	N/A	N/A
Peak VO_2_ (mL kg^−1^ min^−1^)	13	294	3.00 (1.92, 4.08) p < 0.000001	60	3	652	4.64 (2.42, 6.87) p < 0.0001	48%
SBP (mmHg)	15	364	−2.54 (−4.34, −0.75) p = 0.006	78	3	652	−3.79 (−6.18, −1.40, p = 0.002)	0%
DBP (mmHg)	14	337	−2.27 (−3.47, −1.06) p = 0.0002	61	3	652	−0.23 (−3.53, 1.55) p = 0.85	68%
FBG (mmol L^−1^)	15	378	−0.16 (−0.32, −0.01) p = 0.04	81	2	623	−0.18 (−0.47, 0.25) p = 0.21	0%
HDL (mmol L^−1^)	15	265	0.03 (−0.01, 0.08) p = 0.19	71	2	623	0.14 (0.04, 0.25) p = 0.009	61%
TG (mmol L^−1^)	13	308	−0.21 (−0.29, −0.13) p < 0.00001	57	1	606	**−**0.01 (−0.04, 0.02) p = 0.50	0%
LDL (mmol L^−1^)	2	44	−0.03 (−0.05, −0.00) p = 0.02	0	1	722	−0.30 (−0.61, 0.01) p = 0.06	0%

*N* number of people included in analysis, *BMI* body mass index, *peak VO*_*2*_ peak oxygen consumption, *FBG* fasting blood glucose, *SBP* systolic blood pressure, *DBP* diastolic blood pressure, *FBG* fasting blood glucose, *HDL* high density lipoproteins, *TG* triglycerides, *LDL* low density lipoproteins

#### Cardiovascular outcome measures

Peak VO_2_ was significantly improved, MD 3.00 ml kg^−1^ min^−1^ (95% CI 1.92, 4.08, p < 0.000001), in exercise versus control groups. Systolic blood pressure was significantly reduced, MD −2.54 mmHg (95% CI −4.34, −0.75, p = 0.006), as was diastolic blood pressure, MD −2.27 mmHg (95% CI −3.47, −1.06, p = 0.0002), in exercise compared to control groups (Table 2).

#### Metabolic outcome measures

Fasting blood glucose was significantly reduced, MD −0.16 mmol L^−1^ (95% CI −0.32, −0.01, p = 0.04), in exercise compared to control groups. Triglycerides were significantly improved MD −0.21 mmol L^−1^ (95% CI −0.29, −0.13, p < 0.00001); and LDL cholesterol was significantly improved MD −0.03 mmol L^−1^ (95% CI −0.05, −0.00, p = 0.02) in exercise versus control participants. HDL cholesterol was unchanged in exercise versus control participants (Table 2).

### Combined aerobic and resistance exercise versus control

In the comparison of combined exercise versus control participants only: waist circumference, MD −3.80 cm (95% CI −5.65, −1.95, p < 0.0001); peak VO_2_, MD 4.64 ml.kg^−1^ min^−1^ (95% CI 2.42, 6.87, p < 0.0001); systolic blood pressure, MD −3.79 mmHg (−6.18, −1.40, p = 0.002); and, HDL cholesterol, MD 0.14 mmol L^−1^ (95% CI 0.04, 0.25, p = 0.009) were significantly changed in combined exercise versus control groups (Table 2). We did not find any statistically significant benefit of combined exercise over aerobic exercise.

### Comparison of outcome measures according to exercise intensity and type

#### Body composition outcome measures

For the analyses of BMI, body mass, total fat mass and waist circumference, we found no statistical difference between moderate, vigorous and high intensity aerobic exercise. The same was true of combined exercise in regards to exercise intensity (Table 3).

**Table 3:** Sub analysis of effect of exercise training on MetS by exercise intensity

	Moderate		Vigorous	High	
	Aerobic	Combined	Aerobic	Aerobic	Combined
BMI (kg m^−2^)	−0.34* (−0.55,−0.14)	−0.57* (−0.86, −0.27)	−0.23 (−0.57,0.1)	−0.5* (−0.86,−0.14)	−0.10* (−0.14, −0.06)
Waist circum. (cm)	−0.80* (−1.49, −0.12)	−3.99* (−4.91, −3.06)	−1.59* (−2.39,−0.79)	−3.00* (−4.65,−1.35)	−2.40* (−3.83, −0.97)
Total fat mass (kg)	−1.03* (−1.69, −0.37)	N/A	−2.50* (−4.97, −0.03)	−1.80 (−3.76, 0.16)	N/A
VO_2_ max (mL kg^−1^ min^−1^)	2.52* (0.99, 4.40)	4.83* (1.10, 8.55)	3.20* (1.71, 4.69)	5.5* (1.55, 9.45)	4.20* (1.35, 7.05)
SBP (mmHg)	−3.64 (−9.50, 2.22)	−3.35 (−7.06, 0.37)	−1.33* (−1.88, −0.77)	−6.40* (−11.52, −1.28)	−4.10* (−7.21, −0.99)
DBP(mmHg)	−3.35* (−5.50, −1.19)	−1.68 (−3.96, 0.60)	−1.27 (−2.79, 0.25)	−3.40 (−6.94, 0.14)	1.40* (0.56, 2.24)
FBG (m mol L^−1^)	−0.41* (−0.70, −0.11)	0.00 (−0.45, 0.45)	−0.06 (−0.21, 0.08)	0.20* (0.09, 0.31)	−0.30 (−0.66, 0.06)
HDL (m mol L^−1^)	0.06 (−0.01, 0.13)	0.10* (0.04, 0.16)	−0.02 (−0.05, 0.01)	0.16* (0.07, 0.25)	0.21* (0.09, 0.33)
TG (m mol L^−1^)	−0.18* (−0.28, −0.09)	−0.01* (−0.04, 0.02)	−0.25* (−0.42, −0.08)	−0.50* (−0.94, −0.06)	N/A

Data is MD (95% CI)

* p < 0.05

*BMI* body mass index, *VO*_*2*_ *peak* maximal oxygen consumption, *SBP* systolic blood pressure, *DBP* diastolic blood pressure, *FBG* fasting blood glucose, *HDL* high density lipoprotein cholesterol, *TG* triglycerides

#### Cardiovascular outcome measures

Change in peak VO_2_ was highest with high intensity aerobic exercise, MD 5.50 mL kg^−1^ min^−1^, and combined exercise at moderate intensity, MD 4.83 ml kg^−1^ min^−1^. Change in systolic blood pressure was greatest with high intensity aerobic exercise, MD −6.40 mmHg. The change in diastolic blood pressure was similar across all exercise intensities and types, although only significant in moderate intensity aerobic and high intensity combined exercise (Table 2).

#### Metabolic outcome measures

Fasting blood glucose was only significantly changed with moderate or high intensity aerobic exercise, but the effect size was similar in these two analyses. No differences in effect sizes were observed for any of the lipid measures (Table 2).

### Sub-analyses

We found that the addition or absence of dietary intervention did not significantly affect any outcome measures (see Additional file 1: Table S3). Similarly, we found that neither weekly (see Additional file 1: Table S4) nor total program exercise time (see Additional file 1: Table S5) significantly affected outcome measures. We also found that the inclusion of participants with type II diabetes mellitus who also fit diagnostic criteria for MetS did not significantly affect any of the outcome measures.

### Study quantity

We examined several aspects of study quality of included studies. Median TESTex score was 9 out of 15 (see Additional file 1). The distribution of scores was: 1 study scored 5, 2 studies scored 6, 4 studies scored 7, 4 scored 8, 4 scored 9, 9 scored 10, 3 scored 11, 1 scored 12 and 1 scored 13. The following study quality indicators were completed in 50% or fewer of the studies: allocation concealment, assessor blinding, intention to treat analysis, and, activity monitoring of the control group.

### Publication bias

Funnel (Egger) plots of the primary analyses showed mild to moderate evidence of publication bias as more than one-third of the studies fell outside the funnel plot border.

## Discussion

Our analysis is the first to compare the effects of aerobic, and combined aerobic and resistance, exercise on clinical outcome measures in people with metabolic syndrome. Our analysis is also the first to compare exercise at different intensities. Through pooled data analysis, we have shown that aerobic exercise provides a range of improvements in outcomes related to body composition, cardiovascular health and metabolic profile. Although still beneficial, combined aerobic and resistance exercise appears to offer a narrower scope of benefits compared to aerobic activity alone. In terms of exercise intensity, insufficient data exists to generate statistical power to define the optimal training intensity. Assessment of study quality indicated that some aspects of study design could be enhanced for future studies.

### Body composition outcome measures

The change in body mass index with aerobic exercise, while statistically significant, was small and therefore likely to be clinically insignificant. A recent government report on the health burden of obesity from the Australian institute of health and welfare suggests that a change in BMI of 1 unit (1 kg m^−2^), equivalent to 2–3 kg in most women, or 3–4 kg in most men, is considered a minimum requirement to observe improvements in relative risk of serious illness [27]. The changes in BMI and body mass observed in this analysis did not reach the required threshold to improve health. BMI does not distinguish between lean and fat mass; it may therefore hide any significant changes observed with exercise training in this population as any decrease in fat mass would be met with an increase in lean mass. However, changes in waist circumference were almost 4 cm in combined exercise programs, suggesting that this type of exercise prescription may be optimal for reducing central obesity. The work by Willis et al. [28] suggests that an isolated aerobic exercise program is optimal for reducing fat mass and body mass, while a combined program is needed for increasing lean mass in middle-aged, overweight/obese individuals. It is possible that the clinically insignificant effect of the various exercise regimes utilized in the pooled data is due to an increase in lean body mass, given it is likely that the participants in the intervention groups were severely deconditioned.

### Cardiovascular outcome measures

Peak VO_2_ was significantly improved in both isolated aerobic and combined exercise programs. We could not discern which of these approaches was superior as there was no statistically significant difference between the exercise interventions. The change in peak VO_2_ in both analyses was greater than 1 MET (3.5 mL kg^−1^ min^−1^), which is certainly of clinical significance [29]. Impaired age-predicted peak VO_2_ has been associated with increased risk of mortality in the general population [30] and in several chronic illnesses [31–33], including diabetes [34]. Metabolic syndrome is often the pre-cursor for diabetes. Our analysis also identified beneficial effects of aerobic exercise on both systolic and diastolic blood pressure, while combined exercise showed a benefit in reducing diastolic blood pressure. Previous work has suggested aerobic exercise may be superior to resistance exercise for eliciting anti-hypertensive effects [35, 36].

### Metabolic outcome measures

Our analyses of aerobic, but not combined, exercise training reported small improvements in fasting blood glucose, triglycerides and low density lipoproteins. Combined exercise training elicited small changes in high density lipoprotein cholesterol only. With the exception of triglycerides, which reached a reduction of 13% of the normal level, none of these changes are likely to have reached clinical significance, however may collectively contribute to an overall improved health risk profile when combined with body composition and cardiovascular improvements.

### Effect of exercise training intensity

We found that changes in outcome measures related to body composition were significantly improved for sub-analyses of moderate, vigorous and high intensity training. We were unable to find a statistically significant difference between the exercise intensities. With this in mind, we found it difficult to discern is there was a superior exercise intensity (Table 3). Previous work has shown high intensity exercise to be optimal for the treatment of metabolic syndrome [37] and in other chronic disease populations [38–40].

### Recommendations for optimal exercise prescription

It is important to note that while we could not find statistical difference between analyses of aerobic exercise versus control and combined exercise versus control, some analyses showed a trend towards a larger effect size for some outcome measures. Specifically changes in waist circumference, Peak VO_2_ and systolic blood pressure appear to be optimal with combined exercise. In contrast, change in body mass, diastolic blood pressure appear to be optimal with aerobic exercise. Furthermore, neither weekly and total exercise program duration, nor the addition of dietary intervention, appears to show a clear additional benefit from exercise for people with MetS. We must however note that these comparisons were unlikely to yield significantly meaningful findings due to the current paucity of combined exercise training studies in MetS. Our recommendation is that people with MetS should adhere to current diabetes exercise guidelines [41].

### Roles of hyperglycemia and obesity

According to the international diabetes federation, metabolic syndrome is characterised by at least three of five clinical findings [1]. One of these findings is hyperglycemia. So, if this definition of MetS is primarily based on hyperglycemia, then we showed that exercise elicits favourable glycaemic effects. We are, however, unsure if these small changes are clinically meaningful. In contrast, insufficient data currently exists to establish if exercise exerts a favourable effect on insulin resistance, MetS’ underlying mechanism. Leading on from this, one of the main controversies surrounding the relative importance of the different clinical findings relates to the role of obesity. Our data show significant improvements in both BMI and waist circumference, but again we are unsure if these small changes are clinically meaningful. Another point of controversy relates to clinical outcomes. Most of the studies included in our analysis were of insufficient duration to warrant an analysis of hospitalizations and mortality. Nevertheless, a recent longitudinal study clearly demonstrated that MetS is independently associated with an increased 20-year all-cause mortality [42]. In addition, MetS and all its components are associated with unfavourable cardiovascular changes such as increased arterial stiffness, coronary calcium, diastolic dysfunction and carotid intima media thickness [43–45].

#### Study quality

Median study quality was moderate. Future study designs may be improved by improving assessor blinding, conducting intention to treat analyses and introducing, for the first time methods to monitor physical activity levels in the control group participants. Allocation concealment is notoriously difficult in exercise training studies, as this criterion also scored poorly.

#### Publication bias

Our funnel plot analyses indicate there is a good chance that there are one or more unpublished datasets in existence due to negative trial results. While most trial showed improvements in direct and indirect markers of metabolic syndrome, the effect sizes were often small enough to be considered clinically insignificant. Researchers should remember though that while changes in individual outcome measures may be considered small, collectively the changes in the overall outcome measure profile may be clinically meaningful.

#### Limitations

As with most exercise training research, the available sample size was small and thus limits the generalizability of our results. A number of participants were using medications during the course of the studies. Enrolment in trials of behavioural modification are known to raise subject awareness of interventions such as meditation, weight loss, dietary restriction etc.

## Conclusions

Exercise training produces beneficial changes in body composition, cardiovascular and metabolic outcomes in people with metabolic syndrome. For some outcome measures, isolated aerobic exercise seems to be optimal.

## Additional file

**Additional file 1: Table S1.** Excluded randomized controlled trials. **Table S2**. TESTEX Study Quality Assessment. **Table S3**. Analysis of the effects of exercise and diet on MetS. **Table S4**. Sub analysis of effect of exercise training on MetS by weekly exercise training duration (mins). **Table S5**. Sub-analysis of effects of exercise training on MetS by total exercise program time.
