# Supplementary material for: The effect of exercise training on clinical outcomes in patients with the metabolic syndrome: a systematic review and meta-analysis
Source: Cardiovasc Diabetol. 2017 Aug 30;16:110. doi: 10.1186/s12933-017-0590-y (PMC5577843; doi:10.1186/s12933-017-0590-y)
Supplement: Supplementary file 1 — Additional file 1: Table S1. Excluded randomized controlled trials. Table S2. TESTEX Study Quality Assessment. Table S3. Analysis of the effects of exercise and diet on MetS. Table S4. Sub analysis of effect of exercise training on MetS by weekly exercise training duration (mins). Table S5. Sub-analysis of effects of exercise training on MetS by total exercise program time. [file 12933_2017_590_MOESM1_ESM.docx]

**Additional Files**

**PubMed Search Strategy**

| [#](http://www.ncbi.nlm.nih.gov/pubmed/?querykey=8&dbase=pubmed&querytype=eSearch&)11 | | Search (Exercise/Broad[filter]) AND (#10) |  |
| --- | --- | --- | --- |
| [#](http://www.ncbi.nlm.nih.gov/pubmed/?querykey=6&dbase=pubmed&querytype=eSearch&)10 | | Search #8 OR #9 |  |
| [#](http://www.ncbi.nlm.nih.gov/pubmed/?querykey=5&dbase=pubmed&querytype=eSearch&)9 | | Search #1 OR #2 OR #3 OR #4 OR #5 OR #6 OR #7 OR #8 |  |
| [#](http://www.ncbi.nlm.nih.gov/pubmed/?querykey=4&dbase=pubmed&querytype=eSearch&)8 | | Search (((exercise[MeSH Terms]) OR exercise therapy [MeSH Terms]) OR aerobic exercise[Text Word]) OR exercise training[Text Word] OR resistance training [Text Word] OR Weight lifting [Text word] OR Strength Endurance [Text Word] OR Fitness [Text Word] OR Cardiovascular training [Text Word] OR Walk* [Text Word] OR Exertion [Text Word] OR Muscular exercise [Text Word] |  |
| [#](http://www.ncbi.nlm.nih.gov/pubmed/?querykey=3&dbase=pubmed&querytype=eSearch&)7 | | Search ((physical activity [MeSH Terms]) OR physical training [Text Word]) |  |
| [#](http://www.ncbi.nlm.nih.gov/pubmed/?querykey=2&dbase=pubmed&querytype=eSearch&)6 | | Search ((((lifestyle therapy [MeSH Terms]) OR exercise training [Text Word]) |  |
| [#](http://www.ncbi.nlm.nih.gov/pubmed/?querykey=1&dbase=pubmed&querytype=eSearch&)5 | Search (((((insulin resistance [MeSH Terms]) OR exercise training [Text Word]) | |  |
| [#](http://www.ncbi.nlm.nih.gov/pubmed/?querykey=1&dbase=pubmed&querytype=eSearch&)4 | Search (((((overweight [MeSH Terms]) OR exercise training [Text Word]) | |  |
| #3 | Search (((((obese [MeSH Terms]) OR exercise training [Text Word]) | | |
| [#](http://www.ncbi.nlm.nih.gov/pubmed/?querykey=1&dbase=pubmed&querytype=eSearch&)2 | Search (((((diabetes [MeSH Terms]) OR exercise training [Text Word]) | | |
| [#1](http://www.ncbi.nlm.nih.gov/pubmed/?querykey=1&dbase=pubmed&querytype=eSearch&) | Search (((((metabolic syndrome [MeSH Terms]) OR exercise training [Text Word]) | | |

**Table S1. Excluded randomized controlled trials.**

| **Article** | **Reason** |
| --- | --- |
| Bonfanti, 2014 | Article in Spanish |
| Bosak 2009 | Study Design (No Exercise Intervention) |
| Cohen 2008 | Yoga |
| Corey 2014 | Yoga |
| Dash 2014 | Rationale |
| DeVallance 2016 | Resistance Training |
| Dieli-Conwright 2014 | Study Design |
| Gomez-Huelgas 2015 | Study Design |
| Kanya 2014 | Yoga |
| Lee 2012 | Study Design |
| Luley 2014 | Study Design |
| Maki-Nunes | Study Design (Diet and Exercise vs Control) |
| Malin 2012 | Study Design |
| Nanri 2012 | No Exercise Intervention |
| Neumayr 2014 | Study Design |
| Ortega 2016 | Study Design |
| Perez-Idarraga 2015 | Article in Spanish |
| Plaisance 2008 | Acute Measurements |
| Weinheimer 2012 | Study Design (No Exercise Intervention) |
| Zhang 2011 | Study Design (No Exercise Intervention) |

**Table S2. TESTEX Study Quality Assessment**

| Study name | Eligibility criteria specified | Randomly allocated participants | Allocation concealed | Groups similar at baseline | Assessors blinded | Outcome measures assessed >85% of participants | Intention to treat analysis | Reporting of between group statistical comparisons | Point measures & measures of variability reported | Activity monitoring in control group | Relative exercise intensity review | Exercise volume and energy expended | Overall TESTEX |
| --- | --- | --- | --- | --- | --- | --- | --- | --- | --- | --- | --- | --- | --- |
| Babaei (2013) | YES | YES | UNCLEAR | YES | UNCLEAR | YES (1) | NO | YES (2) | YES | NO | NO | YES | 8 |
| Balducci (2010) small | YES | YES | UNCLEAR | YES | YES | YES (3) | NO | YES (2) | NO | NO | YES | YES | 11 |
| Balducci (2010) large | YES | YES | YES | YES | NO | NO (2) | NO | YES (2) | YES | NO | YES | NO | 10 |
| Casella-Filho (2011) | YES | NO | NO | NO | UNCLEAR | YES (1) | NO | YES (2) | YES | NO | YES | NO | 6 |
| Courteix (2015) | YES | YES | UNCLEAR | YES | YES | YES (2) | NO | YES (2) | YES | NO | YES | YES | 11 |
| Damirchi (2014) | YES | NO | YES | YES | UNCLEAR | NO (2) | NO | YES (2) | YES | NO | NO | YES | 9 |
| Donley (2014) | YES | NO | UNCLEAR | NO | UNCLEAR | YES (1) | NO | YES (2) | YES | UNCLEAR | YES | YES | 7 |
| Gonzalez (2014) | YES | NO | YES | YES | UNCLEAR | NO (1) | NO | YES (2) | YES | NO | NO | YES | 8 |
| Irving (2008) | YES | YES | UNCLEAR | YES | UNCLEAR | YES (1) | NO | YES (2) | YES | NO | YES | YES | 9 |
| Irving (2009) | YES | YES | UNCLEAR | YES | UNCLEAR | YES (2) | NO | YES (2) | YES | NO | YES | YES | 10 |
| Kim (2011) | YES | YES | YES | YES | NO | NO (2) | NO | YES (2) | YES | NO | NO | YES | 10 |
| Mager (2008) | YES | YES | UNCLEAR | NO | UNCLEAR | YES (1) | NO | NO | YES | NO | NO | YES | 5 |
| Maresca (2013) | YES | YES | UNCLEAR | YES | UNCLEAR | YES (1) | NO | YES (2) | YES | UNCLEAR | NO | YES | 8 |
| Martins (2016) | YES | NO | NO | NO | NO | YES (1) | NO | YES (2) | YES | NO | YES | NO | 6 |
| Oh (2008) | YES | YES | UNCLEAR | YES | UNCLEAR | YES (1) | NO | YES (2) | NO | NO | NO | YES | 7 |
| Oh (2010) | YES | YES | YES | YES | NO | NO (1) | NO | YES (2) | YES | NO | YES | YES | 10 |
| Oh (2010) | YES | YES | YES | YES | UNCLEAR | NO (1) | YES | YES (2) | YES | NO | NO | YES | 10 |
| Okura (2007) | YES | NO | UNCLEAR | YES | UNCLEAR | YES (1) | NO | YES (2) | YES | UNCLEAR | YES | NO | 7 |
| Reseland (2001) | YES | YES | UNCLEAR | YES | UNCLEAR | YES (2) | NO | YES(2) | YES | NO | NO | NO | 8 |
| Seligman (2011) | YES | YES | YES | YES | YES | YES(2) | NO | YES (2) | YES | YES | YES | YES | 13 |
| Serrano-Ferrer (2016) | YES | YES | YES | NO | UNCLEAR | YES (2) | NO | YES (2) | YES | UNCLEAR | YES | YES | 10 |
| Soares (2014) | YES | NO | YES | YES | UNCLEAR | NO (2) | NO | YES (2) | YES | YES | NO | NO | 9 |
| Sonnenschein (2011) | YES | YES | YES | YES | NO | YES (1) | NO | YES (2) | YES | NO | YES | YES | 10 |
| Stensvold (2010) | YES | YES | YES | YES | NO | YES (3) | NO | YES (2) | YES | NO | YES | YES | 12 |
| Straznicky (2010) | YES | YES | YES | YES | NO | YES (1) | NO | YES (2) | YES | NO | YES | YES | 10 |
| Tjonna (2008) | YES | YES | UNCLEAR | YES | UNCLEAR | YES (3) | NO | YES (2) | YES | NO | YES | YES | 11 |
| Troseid (2004) | YES | YES | UNCLEAR | YES | UNCLEAR | YES (1) | NO | YES (2) | YES | NO | NO | NO | 7 |
| Vargas (2013) | YES | YES | YES | YES | NO | YES (1)? | NO | YES (2) | YES | NO | NO | YES | 9 |
| **TOTALS** | 28 | 21 | 14 | 23 | 3 | 21 (2) | 0 | 27 | 26 | 2 | 16 | 21 | **Median**  **9** |

**Table S3.Analysis of the effects of exercise and diet on MetS.**

|  |  | |
| --- | --- | --- |
| **Outcome Variable** | **Exercise vs Control** | **Diet + Exercise vs Diet only** |
| BMI (kg.m^-2^) | -0.24 [-0.38, -0.10]* | -0.40 [-0.56, -0.24]* |
| Body Weight | -1.16 [-1.89, -0.43]* | -1.30 [-3.59, 0.99] |
| Waist circum. (cm) | -1.27 [-1.95, -0.59]* | -3.10 [-5.82, -0.38]* |
| Hip-to-Waist Ratio | -0.01 [-0.05, 0.03] | -0.01 [-0.04, 0.02] |
| Total Fat Mass (kg) | -1.15 [-1.78, -0.52]* | -1.70 [-3.93, 0.53] |
| VO2 Max (ml.kg^-1^.mim^-1^) | 2.90 [1.60, 4.20]* | 3.92 [1.29, 6.56]* |
| SBP (mmHg) | -3.43 [-5.53, -1.34]* | 1.02 [-3.30, 5.34] |
| DBP(mmHg) | -2.59 [-4.01, -1.18]* | -1.27 [-3.91, 1.38] |
| FBG (m.mol.L^-1^) | -0.20 [-0.38, -0.01]* | 0.01 [-0.33, 0.36] |
| HDL (m.mol.L^-1^) | 0.04 [-0.02, 0.09] | 0.02 [-0.05, 0.09] |
| TG (m.mol.L^-1^) | -0.23 [-0.34, -0.11]* | -0.23 [-0.41, -0.04]* |
| TC (m.mol.L^-1^) | -0.23 [-0.34, -0.13]* | 1.81 [-3.38, 7.00] |
| MetS Diagnosis | 0.12 [0.04, 0.36]* | 0.14 [0.03, 0.77]* |

**Table S4. Sub analysis of effect of exercise training on MetS by weekly exercise training duration (mins)**

|  | <140mins/wk | | 140-200 mins/wk | | >200mins/wk |
| --- | --- | --- | --- | --- | --- |
| **Outcome** | **Aerobic** | **Combined** | **Aerobic** | **Combined** | **Aerobic** |
| BMI (kg.m^-2^) | -0.49 (-0.74, -0.24)* | -0.10 (-0.14, -0.06)* | -0.20 (-0.36, -0.05)* | -0.57 (-0.86, -0.27)* | -0.51 (-1.01, -0.01)* |
| Body Weight | -1.98 (-2.80, -1.16)* | N/A | -0.38 (-1.66, 0.91) | N/A | -1.12 (-2.41, 0.16) |
| Waist circum. (cm) | -1.78 (-2.67, -0.89)* | -2.40 (-3.83, -0.97) * | -1.25 (-2.29, -0.21)* | -4.87 (-6.54, -3.20) * | -0.97 (-2.61, 0.68) |
| Total Fat Mass (kg) | -1.80 (-3.76, 0.16) | N/A | -1.05 (-1.84, -0.25)* | N/A | -1.34 (-2.56, -0.11)* |
| VO2 Max (ml.kg^-1^.mim^-1^) | 2.90 (1.06, 4.75)* | 4.20 (1.35, 7.05) * | 3.56 (0.60, 6.52)* | 4.83 (1.10, 8.55) * | 2.05 (0.80, 3.30)* |
| SBP (mmHg) | -2.08 (-4.72, 0.57) | -4.10 (-7.21, -0.99) * | 0.22 (-4.10, 4.54) | -3.35 (-7.06, 0.37) | -5.03 (-7.84, -2.22)* |
| DBP(mmHg) | -3.95 (-6.94, -0.96)* | 1.40 (0.56, 2.24) * | -0.32 (-1.62, 0.97) | -1.68 (-3.96, 0.60) | -3.40 (-5.78, -1.03)* |
| FBG (m.mol.L^-1^) | -0.23 (-0.61, 0.15) | -0.30 (-0.66, 0.06) | -0.23 (-0.92, 0.45) | 0.00 (-0.45, 0.45) | -0.14 (-0.28, -0.00)* |
| HDL (m.mol.L^-1^) | 0.11 (0.05, 0.17)* | 0.21 (0.09, 0.33) * | -0.01 (-0.06, 0.03) | 0.10 (0.04, 0.16)* | -0.03 (-0.06, 0.01) |
| TG (m.mol.L^-1^) | -0.14 (-0.28, 0.01) | N/A | -0.16 (-0.26, -0.07)* | N/A | -0.41 (-0.55, -0.28)* |
| TC (m.mol.L^-1^) | -0.13 (-0.40, 0.15) | N/A | -0.24 (-0.37, -0.12)* | N/A | N/A |

**Table S5. Sub-analysis of effects of exercise training on MetS by total exercise program time.**

|  | <2000 minutes | | 2000-5000 minutes | >5000 minutes | |
| --- | --- | --- | --- | --- | --- |
| **Outcome** | **Aerobic** | **Combined** | **Aerobic** | **Aerobic** | **Combined** |
| BMI (kg.m^-2^) | -0.33 [-0.64, -0.02]* | -0.10 [-0.14, -0.06]* | -0.44 [-0.78, -0.11]* | -0.26 [-0.54, 0.01] | -0.57 [-0.86, -0.27]* |
| Body Weight | -2.09 [-3.00, -1.19]* | -0.30 [-0.94, 0.34] | -1.24 [-2.21, -0.28]* | -0.13 [-1.79, 1.53] | 0.20 [-0.36, 0.76] |
| Waist circum. (cm) | -1.95 [-2.94, -0.96]* | -2.40 [-3.83, -0.97]* | -0.97 [-1.86, -0.09]* | -1.14 [-2.99, 0.71) | -4.87 [-6.54, -3.20]* |
| Total Fat Mass (kg) | -1.76 [-3.23, -0.28]* | N/A | -1.34 [-2.56, -0.11]* | -0.95 [-1.80, -0.10] | N/A |
| VO2 Max (ml.kg^-1^.mim^-1^) | 3.90 [2.45, 5.34]* | 4.20 [1.35, 7.05]* | 1.92 [0.83, 3.02]* | 3.91 [-1.27, 9.10] | 4.83 [1.10, 8.55]* |
| SBP (mmHg) | -1.88 [-3.85, 0.08] | -4.10 [-7.21, -0.99]* | -5.03 [-7.84, -2.22]* | 1.66 [-5.47, 8.79] | -3.35 [-7.06, 0.37]* |
| DBP(mmHg) | -2.51 [-4.88, -0.13]* | 1.40 [0.56, 2.24]* | -3.06 [-5.12, -1.00]* | -0.31 [-2.12, 1.50] | -1.68 [-3.96, 0.60] |
| FBG (m.mol.L^-1^) | -0.07 [-0.29, 0.14] | -0.30 [-0.66, 0.06] | -0.27 [-0.49, -0.04]* | N/A | 0.00 [-0.45, 0.45] |
| HDL (m.mol.L^-1^) | 0.06 [-0.00, 0.11] | 0.21 [0.09, 0.33]* | -0.03 [-0.06, 0.01] | N/A | 0.10 [0.04, 0.16]* |
| TG (m.mol.L^-1^) | -0.14 [-0.23, -0.05]* | N/A | -0.34 [-0.48, -0.20]* | N/A | N/A |
